# Schistosomiasis Research in the Dongting Lake Region and Its Impact on Local and National Treatment and Control in China

**DOI:** 10.1371/journal.pntd.0001053

**Published:** 2011-08-30

**Authors:** Donald P. McManus, Darren J. Gray, Allen G. Ross, Gail M. Williams, Hong-Bin He, Yue-Sheng Li

**Affiliations:** 1 Division of Infectious Diseases, Queensland Institute of Medical Research, Brisbane, Australia; 2 School of Public Health, Griffith University, Meadowbrook, Australia; 3 School of Population Health, University of Queensland, Brisbane, Australia; 4 Hunan Institute of Parasitic Diseases, Hunan, People's Republic of China; National Institute of Parasitic Diseases China CDC, China

## Abstract

Schistosomiasis is a chronic and debilitating parasitic disease that has often been neglected because it is a disease of poverty, affecting poor rural communities in the developing world. This is not the case in the People's Republic of China (PRC), where the disease, caused by *Schistosoma japonicum*, has long captured the attention of the Chinese authorities who have, over the past 50–60 years, undertaken remarkably successful control programs that have substantially reduced the schistosomiasis disease burden. The Dongting Lake region in Hunan province is one of the major schistosome-endemic areas in the PRC due to its vast marshland habitats for the *Oncomelania* snail intermediate hosts of *S. japonicum.* Along with social, demographic, and other environmental factors, the recent completion and closure of the Three Gorges dam will most likely increase the range of these snail habitats, with the potential for re-emergence of schistosomiasis and increased transmission in Hunan and other schistosome-endemic provinces being a particular concern. In this paper, we review the history and the current status of schistosomiasis control in the Dongting Lake region. We explore the epidemiological factors contributing to *S. japonicum* transmission there, and summarise some of the key research findings from studies undertaken on schistosomiasis in Hunan province over the past 10 years. The impact of this research on current and future approaches for sustainable integrated control of schistosomiasis in this and other endemic areas in the PRC is emphasised.

## Introduction

Schistosomiasis, caused by *Schistosoma japonicum*, disabled and killed millions of Chinese before the national control program for the People's Republic of China (PRC) commenced in the 1950s. This program proved very effective over the subsequent four decades and substantially reduced the disease burden [1]. In 1992, the World Bank provided the PRC a 10-year (1992–2001) US$71 million loan (complemented with US$82 million from the Chinese government) to further boost schistosomiasis control efforts [2], [3]. This is the largest schistosomiasis control project funded to date and, as a result, the number of infected people was reduced to under 1 million by 2001 [3]–[6].

With the termination of The World Bank Loan Project (WBLP) in 2001, however, the steady reduction in the prevalence of and morbidity due to *S. japonicum* came to a halt and, within a short time span, there were again clear signs of re-emergence of the disease in the lake and marshland areas of southern China [4], [7]. Thus, schistosomiasis control was reinstated as a national priority [8], with a long-term national program aimed at reducing human infection and prevalence to less than 5% by 2008 and then below 1% by 2015 in all endemic areas [9], [10].

The Dongting Lake region in Hunan province is one of the major schistosome-endemic areas in the PRC. The marshland habitats for the snail intermediate host (*Oncomelania hupensis hupensis*) of *S. japonicum* are vast, estimated at 1,768 km^2^ in 1996; these are increasing at a rate of 34.7 km^2^ annually mainly due to high silt deposition in the lake from the Yangtze River [11]. It is also anticipated that the Three Gorges dam (TGD) across the Yangtze River will further extend the range of the snail habitats [5], [12], thereby increasing the potential for increased rates of transmission. In many areas, human re-infection, even after extensive drug (praziquantel) treatment, still remains unacceptably high (up to 20% of those treated are re-infected annually due to occupational—mainly fishing—water contact) [11], [13]. In this paper, we review the history and the current status of schistosomiasis control in the Dongting Lake region. We explore the epidemiological factors contributing to *S. japonicum* transmission, and provide insight into future approaches for control that might finally lead to the elimination of schistosomiasis from this focus and other endemic areas in the PRC.

## Methods

We conducted a thorough literature search in NCBI PubMed, the Chinese literature, and personal archives for research articles on schistosomiasis in the Dongting Lake region, with particular emphasis on research undertaken over the last 10 years. The article complements and extends our earlier review on the epidemiology, morbidity, and strategies for control of *S. japonicum* in the Dongting Lake region [11], and that of Yuan et al. [3] on the outcome and prospective of the WBLP schistosomiasis research initiative, which together cover more than 20 years of schistosomiasis research in the PRC.

## The Geography of Dongting Lake

**Figure 1:**
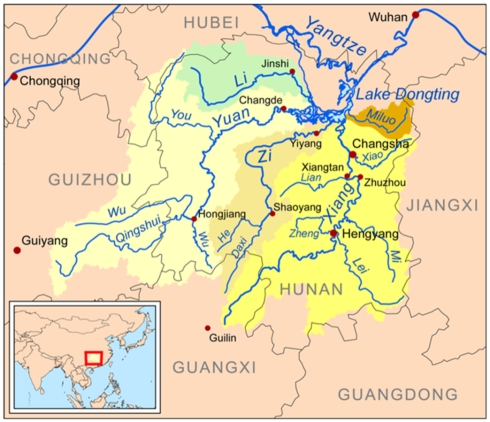
Map of Dongting Lake (a flood-basin of the Yangtze River), Hunan province, People's Republic of China, and its five feeder rivers.

Dongting Lake is located at 28°30′–30°20′ N and 111°40′–113°40′ E in the northeastern part of Hunan province, PRC, and covers a water surface area of 2,681 km^2^ (Figure 1). The lake region is 25–50 metres above sea level and has a warm climate with abundant rainfall. The lake plays an important role in regulating the amount of water in the Yangtze River. It collects the water of four rivers running from upstream into the Yangtze River and stores water when the Yangtze is in flood. The lake is shallow and consists of east, south, and west sections. Its annual water level changes by approximately 15 metres, rising in summer and falling in winter. The water surface area of the lake has slowly decreased due to silt deposition at the margins from connecting rivers in Hunan province, including the Yangtze, and the construction of embankments in the Dongting Lake region.

The rich sediment of the marshland attracted farmers, and several embankments were built to keep out the Yangtze River and to gain more farmland. Unfortunately, silting of mud and sand in the lake, in addition to the anthropogenic environmental transformations in the lowland areas, reduced the lake area and its storage capacity and caused rapid deterioration of the lake's flood diversion and flood storage functions. This diminishing capacity increased the occurrence of disastrous flooding, mainly because of the rupture of embankments. After a serious and major flood in 1998, the State Council of the PRC formulated a policy, the Return Land to Lake Program, to prevent flooding. This program envisioned returning cultivated lands into lake areas with the result that over 1 million people and their domestic animals from schistosomiasis-endemic areas were resettled to newly established towns around Dongting Lake during the period 1999–2004 [12].

## History of Schistosomiasis in the Dongting Lake Region

Archaeological studies first revealed schistosome eggs in a female corpse from Hunan province dating back over 2,100 years [11], [14]. The first clinical case was reported by the American physician O. T. Logan in 1905 [15]. From 1956 to 1980, the Lake Dongting control program focused on snail control and limited drug treatment of patients. Although the number of cases dropped, snail control met with limited success [16], [17]. During the 1980s, funds for schistosomiasis control became severely reduced so that the disease became resurgent, with increasing numbers of chronic cases and outbreaks of acute schistosomiasis, even in cities [13], [18]. Since the establishment in 2004 of an integrated national program of schistosomiasis control, the situation in the Dongting Lake region has improved [19]. By 2010, both human and bovine prevalence had been reduced to less than 5%, with the estimated number of schistosomiasis cases reduced also to 95,000 (Table 1) [20].

**Table 1:** The number of endemic villages, the at-risk population, and the number of schistosomiasis cases over time around Dongting Lake.

Year	Number of Endemic Villages	At-Risk Human Population	Estimated Number of Human Schistosomiasis Cases
1990	2,211	3,081,020	275,746
2000	2,350	3,439,088	205,745
2010	1,735	2,663,458	94,811

1990–2000: Control of schistosomiasis involved human treatment and snail elimination.

2000–2010: Control of schistosomiasis involved annual mass treatment of humans and bovines, health education, and environmental modification.

## The Epidemiology of Schistosomiasis in the Dongting Lake Region

Biological, ecological, social, and economic factors involving interaction between the various hosts, life cycle stages, and the external environment constitute a complicated process in the epidemiology and transmission of schistosomiasis. Transmission patterns in the Dongting Lake region comprise two distinct peaks in the spring/early summer and autumn. Infection starts in April, coinciding with the commencement of the annual rainy season; spring is favourable for *Oncomelania* snails as the water levels rise in the lake and the water temperature increases [11]. The first peak in *S. japonicum* transmission is from May to early July, when the highest numbers of snails are infected. When the water level reaches its maximum in mid-July/September, the older snails die off, the density of cercariae is reduced, and although transmission still occurs, it is reduced to quite low levels [11], [12]. The second peak in transmission occurs from October to November [11], [12]. The end of the transmission season in the Dongting Lake region coincides with the onset of cold weather in late autumn/early winter and the dry season, when the lake water levels recede and the snails bury themselves underground in order to survive [11], [12].

**Figure 2:**
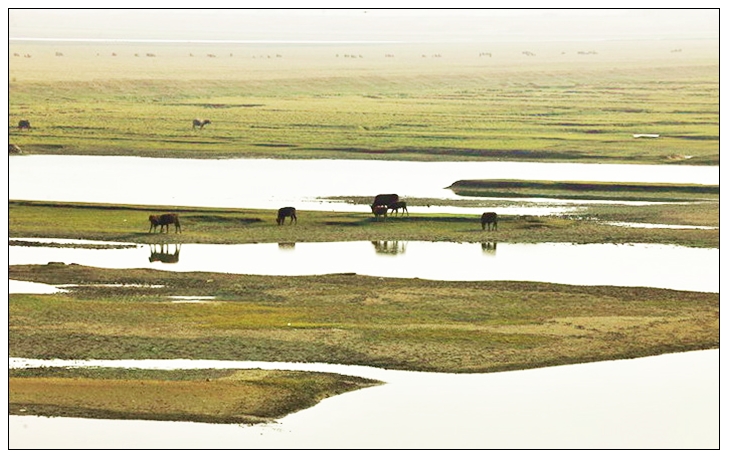
Water buffaloes (a major transmission source of *Schistosoma japonicum*) grazing on part of the vast marshlands (*Oncomelania* snail host habitats) in the Dongting Lake area.

Spatial modelling of the *S. japonicum* infection risk among humans in the Dongting Lake region suggests that socio-demographic (i.e., age, sex) and economic factors (i.e., occupation) may be more important than environmental factors in explaining the spatial distribution of *S. japonicum* there [21]. In addition, the presence of infected livestock poses an increased risk. Both humans and animals act as definitive hosts for *S. japonicum*. This zoonotic feature impacts on the epidemiology of schistosomiasis japonica in the Dongting Lake region and makes disease control more complicated [10], [22], [23]. It is now well established that bovines, particularly water buffaloes (*Bubalus bubalis*), play a major role in the transmission of *S. japonicum* (Figure 2) [24], [25].

## Impact of the Three Gorges Dam on *S. japonicum* Transmission in the Dongting Lake

The TGD is the world's largest hydroelectric project, and by 2009 it was generating 18,600MW of power for the whole of the PRC. The dam will also help to control the lower Yangtze floods, which are much feared. The 2,300-m long, 185-m high dam has resulted in the creation of a 630-km long reservoir that has inundated 115,000 acres of cultivated land, requiring the resettlement of approximately 2 million people. The dam is predicted to change water and sand distribution downstream, impacting Dongting Lake and other Yangtze River areas, with the distribution and numbers of schistosome-infected *Oncomelania* snails altered, and increasing transmission of *S. japonicum* and its re-introduction into previously controlled areas [26]–[30].

## Pathology and Morbidity due to Schistosomiasis in the Dongting Lake Region

The major pathological lesions of *S. japonicum* infection are the granulomatous reactions formed in the liver in response to trapped parasite eggs [10], [31]. Clinical manifestations of *S. japonicum* infection can be divided into three forms: acute, chronic, and advanced hepatic disease [21], [32], [33].

Advanced hepatic disease manifests as two forms—periportal and septal. The former is similar to schistosomiasis mansoni and leads to portal hypertension. The latter is unique to *S. japonicum* and is made worse by habitual alcohol intake. Annual mortality due to schistosomiasis japonica has been estimated to be 0.27% in the Dongting Lake region [11].

Hepatic fibrosis (HF) in schistosomiasis is regulated by cytokines and chemokines [34]. HF development depends, in part, on a major gene locus located on chromosome 6q23. The connective tissue growth factor (*CTGF*) gene is located in this region and encodes a strongly fibrogenic molecule. A recent study [35] showed that two groups of Chinese living in Hunan province (farmers [*n* = 294]; and fishermen [*n* = 300] originating from Jiangsu, Jiangxi, and Hubei provinces, but living on Dongting Lake) had variants of CTGF that are associated with HF due to *S. japonicum* infection [35]. The single nucleotide polymorphism (SNP) rs9402373, which lies close to *CTGF,* was shown to be associated with severe HF in the two groups of Chinese (farmers and fishermen) infected with *S. japonicum*. Furthermore, another SNP (rs12526196), also located close to *CTGF*, was shown to be independently associated with severe fibrosis in the aforementioned Chinese individuals. These variants may be valuable markers for the prediction of disease progression, as they identify a critical step in the development of HF that could be a future target for chemotherapy.

Hepatitis B virus (HBV) infection is a serious health problem worldwide and is highly endemic in the PRC [36]. Co-infection with *S. japonicum* and HBV is associated with accelerated deterioration in hepatic function, often referred to as decompensated liver disease [37]. Data from an extensive study in 2007–2008 showed that co-infection with HBV and *S. japonicum* in 102 patients with hepatosplenomegaly led to more severe fibrosis and inflammatory activity in the liver [32], a feature noted also in an earlier study from this area in the 1990s [38].

Other morbidities associated with schistosome infection, including pulmonary hypertension or cerebral schistosomiasis (neuroschistosomiasis), are uncommon in the Dongting Lake region, where their impact appears limited [10].

## Diagnosis

Current schistosomiasis diagnosis used widely in the Dongting Lake region involves parasitological methods, immunological assays, and morbidity assessment using ultrasonography (US), computerised tomography (CT) scanning, and magnetic resonance imaging (MRI).

### Parasitological Methods

The detection of eggs in faeces is diagnostic for schistosomiasis, and the Kato-Katz (KK) method, being simple, rapid, and inexpensive, has been used extensively in the Dongting Lake region to determine the burden of *S*. *japonicum*. Population-based studies among fishermen and other occupational groups in the lake area have demonstrated that mean egg burdens correlate with the mean severity of disease [39]. As the prevalence and intensity of *S. japonicum* infection has been reduced in Dongting Lake, it has proven inadvisable to continue to use only the KK method for diagnosis. The miracidium-hatching test is a traditional diagnostic method that is now used as a complementary method to determine the presence of an infection [5].

### Immunological Assays

Various commercial immunodiagnostic kits based on serum ELISA and the indirect hemagglutination (IHA) test for detection of anti–*S. japonicum* antibodies have been applied in the Dongting Lake region [32], [40]. Antibody detection is useful in a few specific circumstances, such as when screening for new and re-emerging endemic areas, where the procedure is more sensitive than stool examination. Nevertheless, faecal examination is recommended, if there is positive serology, to increase specificity and because antibodies persist after parasitological cure. A positive serological test may be an early indication of infection in patients where no eggs are present, such as those with acute schistosomiasis (Katayama syndrome). Serology may also be informative in monitoring and determining whether infection has re-emerged in a region after an apparently successful control program [41].

The detection of non-invasive biochemical markers of hepatic fibrosis has been a focus of research on Dongting Lake patients with schistosomiasis. Serum levels of procollagen peptide (types III and IV), the P1 fragment of laminin, hyaluronic acid, fibrosin, TNF-aR-II and sICAM-1, tissue inhibitors of matrix metalloproteinases (TIMP)-1, and platelet-derived growth factors (PDFG)-BB may be elevated in patients with severe hepatic fibrosis [5], [42]. Another Dongting Lake study determined the diagnostic value of non-invasive serum biomarkers of fibrosis using 84 advanced schistosomiasis japonica patients, recruited at different stages of disease progression, and nine controls, histologically assessed by wedge liver biopsies [43]. Aspartate aminotransferase and the platelet ratio index levels were reliable and sensitive markers for differentiating significant hepatic fibrosis in these advanced schistosomiasis patients, whereas serum hyaluronic acid and TIMP-1 showed potential as additional markers for the diagnosis of fibrosis and cirrhosis [43].

### Morbidity Assessment

Ultrasonography (US) is a safe, rapid, non-invasive, and relatively inexpensive technique for assessing schistosomiasis-related lesions in individual patients and in community surveys [44], [45]. US has been used extensively for classifying morbidity/liver fibrosis in schistosomiasis patients from Dongting Lake and other endemic areas of the PRC using World Health Organization criteria [4], [5], [13], [35], [46], [47]. These criteria were improved following hepatosplenic measurements among 550 Chinese individuals, aged 3–59 years, from Yueyang city, a non-endemic area for schistosomiasis around Dongting Lake [46]. A recent study of lake patients, involving a direct comparison of *S. japonicum*–induced fibrosis detected by US and liver biopsy pathology, indicated a moderate agreement between the two procedures, although US underestimated hidden fibrotic lesions in the liver [32].

Clinical CT or MRI scans are not generally used to evaluate morbidity due to *S. japonicum* infection in Hunan province and other parts of the PRC unless a hospitalised patient has neurologic manifestations. This is not uncommon; however, as among groups of Chinese adults hospitalised with schistosomiasis, up to 4.3% have been shown to have central nervous system involvement [48].

## Chemotherapy and Treatment

Praziquantel (PZQ) is the mainstay of community-based schistosomiasis treatment in the Dongting Lake region; however, PZQ has an inherent weakness in that it is unable to prevent re-infection. Routine hospital treatment is 60 mg/kg (in divided doses); for mass field chemotherapy, a single dose (40 mg/kg) is used. The number of people offered chemotherapy in the Dongting Lake region is approximately 400,000–500,000 annually [20], which is substantially more than the actual number of infections confirmed by parasitological or serological examination. However, this approach is unlikely to be sustainable or result in reduced *S. japonicum* prevalence in the long term, and the practice of mass untargeted chemotherapy could lead to the selection of PZQ-resistant parasites. It should be stressed, however, that there is no evidence as yet of PZQ resistance having been reported for *S. japonicum*.

Artemether (AM) is effective against juvenile schistosomes in animals and humans [49], [50], and it has been developed as a prophylactic for the prevention of patent schistosome infections [49]–[51]. A randomised, double-blind, placebo-controlled trial evaluated the efficacy and safety of combined PZQ and AM chemotherapy for acute schistosomiasis japonica in 196 patients living in the Dongting Lake region [52]. The combination chemotherapy did not improve treatment efficacy compared with PZQ alone. However, PZQ given as a dosage of 60 mg/kg (1 day, 3×20 mg/kg doses at 4- to 5-hour intervals), was as effective as a dosage of 120 mg/kg (6 days, 20 mg/kg for each day split into three doses at 4- to 5-hour intervals) that is currently used for treating acute schistosomiasis in PRC [52].

A new initiative was instigated recently in the Dongting Lake region, and other parts of the PRC, for the clinical management of severe cases of hepatosplenic schistosomiasis that involves the surgical removal of the spleen to ameliorate the effects of advanced disease [32]. Currently, there are approximately 6,000 severe cases of hepatosplenic schistosomiasis, which comprise 6.3% of the currently estimated number of patients in the Dongting Lake region; some 1,200 patients with splenomegaly were accepted for this surgery in 2008–2010. Follow-up of this large cohort of patients is planned so that the long-term benefits of this surgery can be assessed. This is important as it has been shown that splenectomy can reduce blood flow to the portal vein by 40%, thereby reducing hypertension in the vein; indeed, over 80% schistosomiasis japonica patients survive for longer than 10 years after splenectomy, with 10% surviving for 20 years following this surgery [53].

## Schistosomiasis Control in the Dongting Lake Region

The control of schistosomiasis in the Dongting Lake region has evolved over past decades from snail control through mollusciciding, then mass chemotherapy on its own, to the current integrated approach, which involves health education, environmental modification, large-scale periodic PZQ treatment of humans and domestic animals—especially cattle and water buffaloes—and focused snail control [54]. An integrated approach is the key to sustainable schistosomiasis control and work done in the Dongting Lake region and throughout the PRC is leading the way [7], [8], [55]. Table 1 shows the impact of integrated control measures in reducing the number of schistosome-endemic villages, the human population at risk, and the estimated number of human schistosomiasis cases between 1990 and 2010 in the Dongting Lake region.

**Figure 3:**
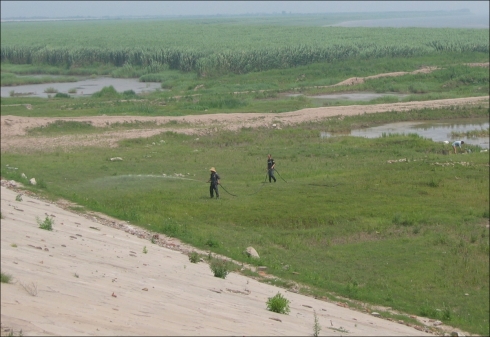
Snail control through mollusciciding (niclosamide, 50% wettable powder, 2 g/M^2^ by spraying), performed annually March to May in the Dongting Lake area.

**Figure 4:**
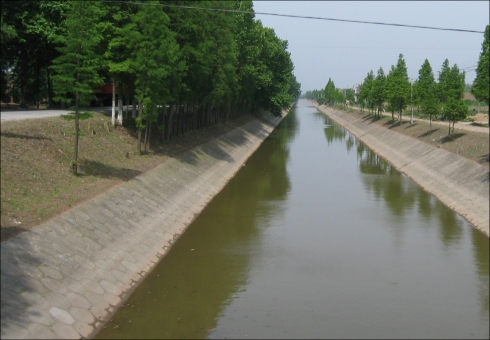
Concrete irrigation system for environmental modification prevents the establishment of *Oncomelania* snail habitats and subsequent schistosome transmission in the Dongting Lake area.

Niclosamide is currently the molluscicide of choice for snail control in the PRC, despite the severe environmental pollution that can result if it is applied indiscriminately. In 2009, it was estimated that a total of 280,000 kg of niclosamide was applied to 11.93 km^2^ of snail habitats in the Dongting Lake region. A new dosing regimen of 25% niclosamide has been shown to exhibit the same snail-killing effect as the conventional 50% [56] (Figure 3). Environmental modification and management are also important for snail control. During the period of 2007–2009, 3.84 km^2^ of *Oncomelania* snail habitats were permanently environmentally modified around the Dongting Lake region (Figure 4).

The provision of good health education and improved human sanitation in endemic areas can ultimately help reduce schistosome transmission. Various types of education materials, including videos and booklets, have been developed and annually distributed in PRC, with very high coverage in school-age children. To test the effectiveness of these materials, 2,870 school children from 80 schools and their parents, residents of the Dongting Lake region, were divided into experimental and control groups [57], [58]. There were significant improvements among the children and their parents in the experimental group in relation to their knowledge of schistosomiasis generally, and in the procedures for screening and treatment of the disease [57], [58]. The longer term benefits of applying such health education packages on a broader scale as a component of an integrated approach for schistosomiasis control need to be evaluated.

Sanitation improvement, facilitated by constructing lavatories and latrines in schistosome-endemic villages, is now a high priority for Hunan province, as there is a direct link between rural economic development and the local peoples' concept of improving health [59], [60]. In recent years, the PRC national and local Hunan governments provided funding to 256,968 households for improving sanitation in *S. japonicum*–endemic villages around the lake [20], so as to facilitate the prospects for sustainable schistosomiasis control.

## Vaccine Development Studies in the Dongting Lake Region

It is difficult and costly to sustain the current control programs for schistosomiasis in the Dongting Lake region and other endemic areas of the PRC, so there is a need for a vaccine for long-term prevention. Bovines (cattle and water buffaloes) are the major reservoirs for *S. japonicum* infection in the PRC, with estimates that 75%–90% of egg contamination comes from this source, reinforcing the rationale for developing a transmission-blocking veterinary vaccine against *S. japonicum* [10], [24], [25], [61]. Even if only partially effective, such a vaccine could be incorporated as part of a multi-component integrated control program [55].

Work undertaken in Hunan province recently evaluated the efficacy of two vaccine candidates (SjCTPI and SjC23) as plasmid DNA vaccines against *S. japonicum* in water buffaloes [62]. Mathematical modelling [62], [63] indicated that either of the two vaccines, in combination with human chemotherapy, could lead to a significant reduction in schistosome transmission. The SjC23 DNA vaccine is currently being field-tested in bovines under natural challenge infection conditions in Anhui Province, and further large-scale field testing in 12 villages endemic for schistosomiasis commenced in 2010 around Dongting Lake as a part of an integrated control package involving bovine vaccination, chemotherapy, and snail control.

## New Control Strategies to Block *S. japonicum* Transmission

**Figure 5:**
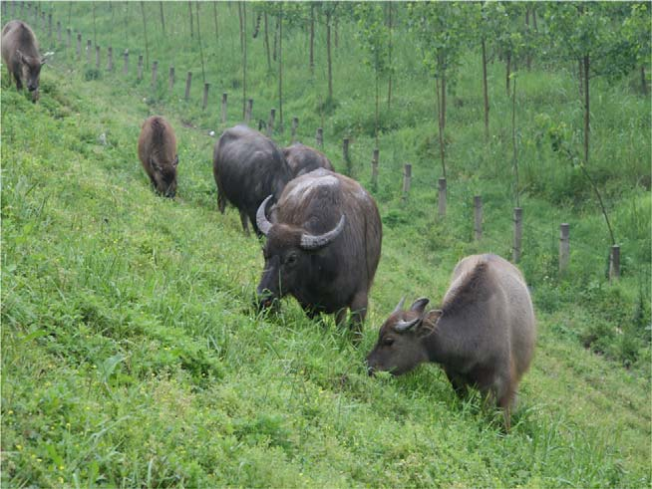
Simple fence to prevent water from buffaloes grazing on the vast marshlands (*Oncomelania* snail host habitat) of the Dongting Lake region.

A revised integrated strategy for schistosomiasis control was proposed in 2004 by the State Council of the PRC and implemented by the Chinese Ministry of Health to limit the contamination of the environment with schistosome eggs by reducing the roles of bovines and humans as sources of infection for snails [64]. The approach emphasises health education, safe water systems, adequate sanitation, and mechanised farm equipment, along with chemotherapy. A special feature was to reduce the number of water buffaloes by replacing them with motorised tractors or to fence them off if farmers showed reluctance to part with their animals. The approach has proved effective in two intervention villages along Poyang Lake in the southeastern province of Jiangxi, where the strategy to reduce the transmission of *S. japonicum* infection from humans and cattle to snails led to a dramatic reduction in the number of infected snails in their natural habitat [64]. Validation of this control strategy in a pilot village (Hongjia Zhou) in the Dongting Lake region through the construction of fences to prevent bovine grazing on snail habitat beach areas similarly resulted in significant reductions in the rates of *S. japonicum* infection in humans and *Oncomelania* snails [65] (Table 2 and Figure 5). A drawback to the approach of replacing livestock with mechanised tractors is that they are expensive to buy and maintain and they are not suitable to all terrains, thus limiting the coverage of such a program.

**Table 2:** Human and bovine *Schistosoma japonicum* infection rates, and snail densities in Hongjia Zhou village, Dongting Lake.

Year	Snail Density (0.11 m^2^)		*S. japonicum* Infection Rates (%)	
	Live Snails	Infected Snails	Humans	Bovines
2004*	0.67	0.0058	11.27	4.90
2005	0.26	0.0017	10.30	4.08
2006	0.14	0	8.26	2.15
2007	0.11	0	3.17	0
2008	0.10	0	3.12	0

*Fences were installed to prevent bovines grazing on *Oncomelania* snail-infested beach areas.

It is estimated that there is a population of 100,000 itinerant fishermen around Donting Lake; 30%–60% are egg-positive by KK, due to their high daily water contact associated with their lifestyle and work commitments [11], [66], [67]. Based on these estimates, the Hunan government commenced in 2008 a new control strategy for these fishermen with the provision of a free land and house package. Several new villages have now been established around Dongting Lake to relocate the floating population of fishermen. No fishing is permitted from January to the end of June; the fishermen work at home or farm, and are offered schistosomiasis treatment. During the fishing season (July to December), the fishermen are allowed to live on their boats and fish. This strategy shortens the annual period of water contact by the fishermen to 6 months, thereby reducing infection and, at the same time, helping to protect the lake from overfishing.

## Influence of Research on the Current Local and National Strategies for the Treatment and Control of Schistosomiasis

**Figure 6:**
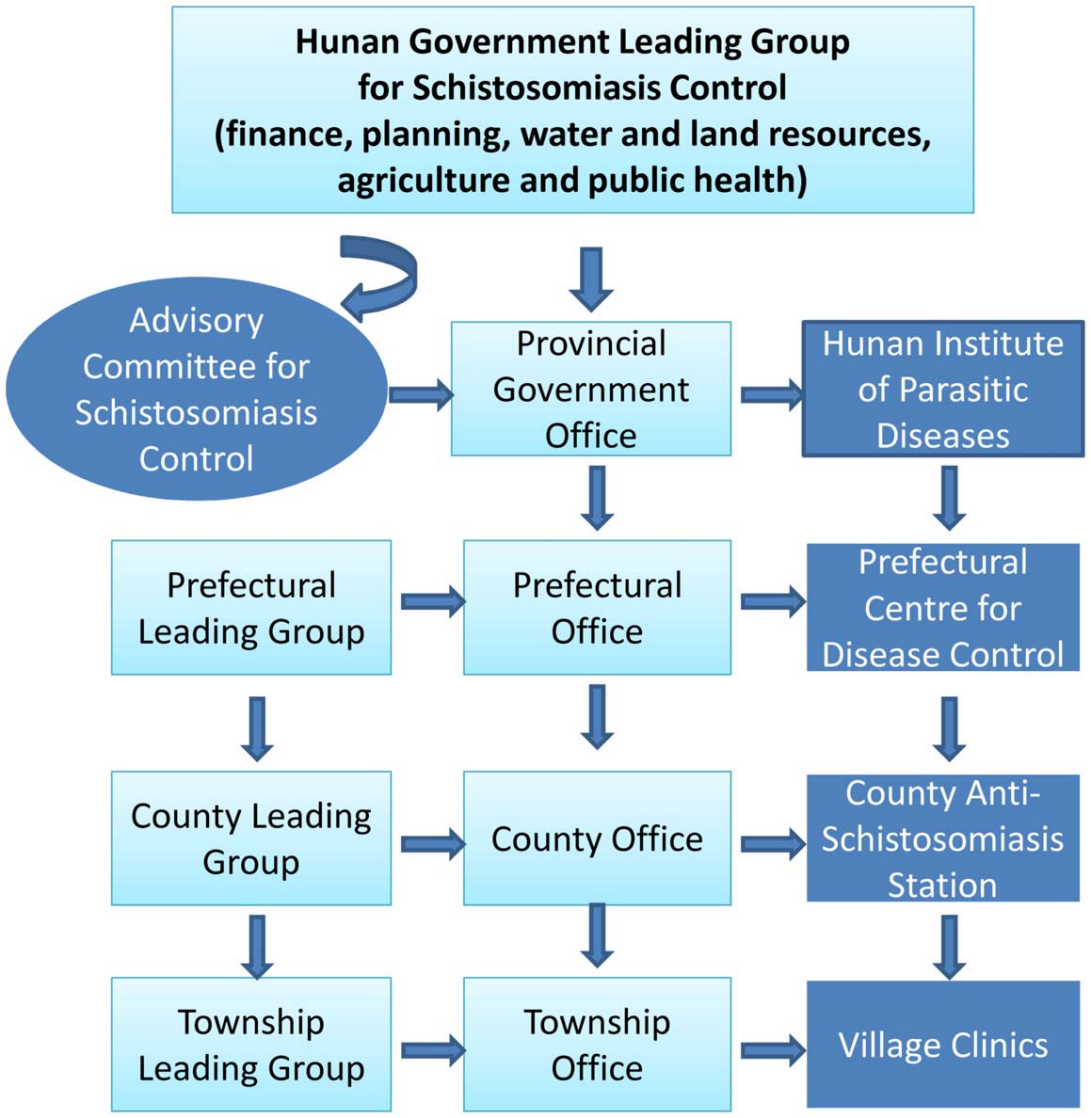
Flowchart of the structure and organisation for schistosomiasis control around the Dongting Lake region.

Currently, approximately 4,000 professional staff are employed at different levels to control schistosomiasis in the Dongting Lake region (Figure 6). An appropriate and stable management for control activities, from provincial to township level, has existed for many years and is considered a crucial factor in the progress achieved to date [11]. This well-developed system makes it possible to organize and carry out routine control activities effectively. Technical support, professional staff training and supervision, evaluation of the control activities, and new implemented approaches are under the auspices of the Hunan Institute of Parasitic Diseases (HIPD), which has over 400 professional staff and a 300-bed hospital for treating patients with schistosomiasis. The contributions of HIPD and its numerous national and international collaborators to schistosomiasis research have been well recognised, and a summary of the impact of some of these key research findings on the current local and national treatment and control options for schistosomiasis is highlighted in Box 1. Some key learning points and publications relevant to this review are shown in Boxes 2 and 3.

**Box 1. The Impact of Research Conducted in the Dongting Lake Region on the Current Local and National Policy for Diagnosis, Treatment, and Control of Schistosomiasis**

**Impact on Schistosome Transmission**

1. Determined *S. japonicum* transmission patterns around the Dongting Lake as a pre-requisite for defining the timing of intervention options for the national control strategy for schistosomiasis.
2. Determined the key role of bovines in schistosome transmission with the result that reducing the rate of transmission of *S. japonicum* infection from water buffaloes and cattle to snails is now one of the major features of the national strategy to control schistosomiasis in China.
3. Carried out spatial modelling of the *S. japonicum* infection risk among humans, which showed that socio-demographic and economic factors may be more important than environmental factors in explaining the spatial distribution of *S. japonicum* in the Dongting Lake area, and probably also in other endemic areas in China.

**Impact on Diagnosis**

1. Demonstrated that a decrease in alcohol intake can reduce the morbidity of chronic and advanced schistosomiasis.
2. Identified genetic markers predicting the severity of disease progression, which could be utilised in future control options for identifying high-risk individuals susceptible to severe schistosomiasis.
3. Confirmed the value of the miracidium-hatching test (MHT) as a complementary method for determining schistosome infection and as a sensitive approach to estimate disease distribution in areas of low infection intensity.
4. Utilised indirect hemagglutination (IHA) for surveillance of schistosomiasis and for monitoring disease re-emergence at the national level.
5. Evaluated non-invasive biochemical markers of hepatic fibrosis and disease progression so as to facilitate appropriate treatment for advanced schistosomiasis.
6. Improved World Health Organization ultrasound criteria for *S. japonicum*–induced disease progression and the classification of morbidity/liver fibrosis so as to provide clinicians with advice on the appropriate treatment of patients with advanced schistosomiasis.

**Impact on Treatment and Control**

1. Demonstrated that co-infection of *S. japonicum* with HBV increases liver fibrosis and inflammatory activity in the liver, with the result that prevention and combined treatment for both infections should be considered as routine for schistosomiasis control.
2. Demonstrated that combined artemether (AM)/praziquantel (PZQ) is not effective for the treatment of acute schistosomiasis, but that PZQ given at a dosage of 60 mg/kg is as effective as the dosage of 120 mg/kg used currently, thereby impacting national policy.
3. Established the use of splenectomy for the clinical management of severe cases of hepatosplenic schistosomiasis.
4. Demonstrated that health education and improved sanitation can play pivotal roles in the national program for the integrated control of schistosomaisis.
5. Pioneered the testing in bovines of a transmission-blocking veterinary vaccine for schistosomiasis as a key intervention in the future national strategy for integrated control.
6. Targeted Dongting Lake fishermen, with very high exposure to *S. japonicum*, for PZQ treatment, and effected changes in their domicile and lifestyle to reduce infection and to help protect the lake from overfishing.

**Box 2. Key Learning Points**

- Dongting Lake is one of the major endemic areas for schistosomiasis in China.
- Transmission in the Dongting Lake region occurs from spring to autumn with two distinct transmission peaks: May to July and September to November.
- Bovines, particularly water buffaloes, are the major reservoir hosts for schistosomiasis in Dongting Lake.
- It is predicted that the recently completed Three Gorges Dam will increase the transmission of schistosomiasis in the Dongting Lake region and re-introduce the disease into previously controlled areas.
- Co-infections of *Schistosoma japonicum* and HBV lead to more severe liver fibrosis and inflammation, although splenectomy can ameliorate the effects of advanced disease.
- Praziquantel (60 mg/kg) is an effective clinical treatment but does not prevent re-infection.
- Multifaceted integrated control—combining human treatment, snail control, health education, improved sanitation, and bovine vaccination—is the key to the elimination of schistosomiasis in the Dongting Lake region.

**Box 3. Key Publications**

- McManus DP, Gray DJ, Li YS, Feng Z, Williams GM, et al. (2010) Schistosomiasis in the Peoples' Republic of China: the era of the Three Gorges Dam. Clin Microbiol Rev 23: 442–466.
- Gray DJ, Williams GM, Li YS, Chen HG, Forsyth SJ, et al. (2009) A cluster-randomised intervention trial against *S. japonicum* in the Peoples' Republic of China: bovine and human transmission. PLoS ONE 4: e5900. doi:10.1371/journal.pone.0005900.
- Da'Dara AA, Li YS, Xiong T, Zhou J, Williams GM, et al. (2008) DNA-based vaccine protects against zoonotic schistosomiasis in water buffalo. Vaccine 26: 3617–3625.
- Hou XY, McManus DP, Gray DJ, Balen J, Luo XS, et al. (2008) A randomised, double-blind, placebo-controlled trial on the safety and efficacy of combined praziquantel and artemether treatment for acute Schistosomiasis japonica in China. Bull World Hlth Org 86: 788–795.
- Yuan LP, Manderson L, Tempongko MS, Wei W, Aiguo P (2000) The impact of educational videotapes on water contact behaviour of primary school students in the Dongting Lakes region, China. Trop Med Int Health 5: 538–544.
- Dessein A, Chevillard C, Arnaud V, Hou X, Zhou J, et al (2009) Variants of CTGF are associated with hepatic fibrosis in Chinese, Sudanese and Brazilians infected with schistosomes. J Exp Med 206: 2321–2328.

## The Challenges Ahead

The current control strategy for schistosomiasis in the Dongting Lake region consists of an integrated approach. Although great strides have been made over the past four decades in the reduction of incidence, prevalence, and intensity of *S. japonicum* infection in the Dongting Lake region, much still needs to be done, as schistosomiasis control requires long-term commitment by the local and national Chinese authorities. Presently, *S. japonicum* infection in humans and water buffaloes has been controlled close to under the targeted 5% level, and the general aim now, in line with other schistosome-endemic areas in the PRC, is to further reduce prevalence and infection to under 1% in the Dongting Lake region by 2015.

Schistosomiasis can be controlled with vigorous political and financial commitment, but in reality, local elimination has proved difficult and prolonged long-term efforts are needed. Despite the integrated control strategy currently in place in the Dongting Lake region, the extensive distribution of snail habitats, and the behaviour and lifestyles of residents, make sustainable schistosomiasis control leading to elimination highly challenging in the foreseeable future. If sustainable control is to become a reality, considerable improvements are necessary in the living environment of Dongting Lake inhabitants, including the provision of safe water supplies and adequate sanitation through inter-sectoral collaboration and local economic development. Additionally, environmental modification, in line with “the new socialist countryside initiative” in the PRC [59], will be required for snail control to block schistosome transmission in combination with the introduction of new environmentally friendly molluscicides and vaccination of bovine reservoir hosts to prevent re-infection of humans and reduce morbidity [55]. Furthermore, community awareness of schistosomiasis and other parasitic diseases can be increased by building on pre-existing knowledge and perceptions. Accordingly, health education will likely play a key role in the future control of schistosomiasis, an approach that will be especially valuable in those Dongting Lake communities with little or no prior knowledge of its prevention or treatment.
